# Insomnia, Fatigue, Bladder Disorders and Mood Disorders among Polish Patients with Multiple Sclerosis: Cross-Sectional Study

**DOI:** 10.3390/jcm13041043

**Published:** 2024-02-12

**Authors:** Michalina Rzepka, Tomasz Chmiela, Aleksandra Kaczmarczyk, Ewa Krzystanek

**Affiliations:** 1 Department of Neurology, Faculty of Medical Sciences in Katowice, Medical University of Silesia, 40-752 Katowice, Poland; tchmiela@sum.edu.pl (T.C.); a30kajkowska@wp.pl (A.K.); 2 Department of Neurology, Faculty of Health Sciences in Katowice, Medical University of Silesia, 40-635 Katowice, Poland; ekrzystanek@sum.edu.pl

**Keywords:** multiple sclerosis, insomnia, anxiety, depression, fatigue, bladder disorders

## Abstract

Background: To investigate the prevalence of sleep disorders in patients with multiple sclerosis (PwMS) in comparison to healthy controls (HCs), we aim to explore the correlation between sleep disorders and fatigue, bladder dysfunction, mood disorders in PwMS. Methods: This study involved 175 PwMS and 115 HCs. We conducted a self-administered survey using questionnaires (the authors’ questionnaire, the Athens Insomnia Scale (AIS), the Epworth Sleepiness Scale (ESS), the Modified Fatigue Impact Scale (MFIS), the Fatigue Severity Scale (FSS), the Hospital Anxiety and Depression Scale (HADS), and the Numerical Rating Scale (NRS). The neurological disability was determined using Expanded Disability Status Scale. Logistic regression was used to estimate odds ratios (ORs) with 95% confidence intervals (CIs). Results: According to AIS, insomnia was found in 20.6% of PwMS compared to 9.6% of HCs (*p* < 0.001). Comparing female and male PwMS, we observed that insomnia was more prevalent among female PwMS (25.95% vs. 4.55%, respectively, *p* < 0.05). Excessive daytime sleepiness was more prevalent in female PwMS (*p* < 0.05). Female PwMS were more fatigue based on the FSS and the MFIS (*p* < 0.05). Bladder disorders were observed in 39.43% of PwMS and were significantly linked to MS (*p* < 0.001). Sleep disturbances were associated with anxiety disorders (OR = 0.22, 95% CI 0.12–0.32 *p* < 0.001), bladder dysfunction (OR = 0.52 95% CI 0.16–0.87 *p* < 0.05), and female gender (OR = 0.49, 95% CI 0.037–0.94 *p* < 0.05). Conclusions: Insomnia is prevalent among PwMS. Our study revealed independent predictors of sleep disturbances among PwMS: female gender, bladder disorders, and anxiety.

## 1. Introduction

Multiple sclerosis (MS) is a chronic inflammatory and demyelinating disease of the central nervous system (CNS) accompanied by a parallel neurodegenerative process [1]. The disease predominantly affects women and is the leading cause of non-traumatic neurological disability among young adults. The prevalence of MS is estimated to be 131.2 per 100,000 inhabitants in Poland and over 2.8 million people (35.9 per 100,000 population) worldwide, with a continuously increasing rate in recent decades [2,3]. Patients with MS (PwMS) can experience a variety of symptoms depending on the location and extent of the CNS damage. Among these symptoms, sleep disorders are often overlooked.

Sleep disorders are pervasive in PwMS and more prevalent compared to the general population [4]. The most common sleep disorder among PwMS is chronic insomnia with an overall prevalence reported between 22 and 52%, depending on the diagnostic tools used in the studies [5]. The etiology of sleep disorders in PwMS may be multifactorial, including primary sleep disorders, such as insomnia, sleep-related breathing disorders (mainly obstructive sleep apnea (OSA)), and restless legs syndrome (RLS). Sleep disorders’ causes may also be secondary to other symptoms of MS spasticity, pain, sensory disturbances, bladder dysfunction, mood disorders, or adverse effects of disease-modifying treatments (DMTs). On the other hand, poor sleep quality may exacerbate debilitating symptoms of MS like fatigue, which affects more than 75% of PwMS or depressive and anxiety disorders [6]. Depressive disorder is the most common psychiatric symptom in MS, with a prevalence up to 50% of patients [7]. Anxiety, which may be overshadowed by depression, can also result in inadequate sleep quality. The overlaps between sleep disorders and fatigue or mood disturbances are estimated to be difficult to distinguish [8]. Bladder dysfunction is observed in up to 80% of PwMS, particularly in those with progressive form of the disease, but it may also occur at the onset of the disease [9]. Bladder disorders in PwMS encompass both storage and voiding symptoms. Storage symptoms consist of overactive bladder syndrome, characterized by urinary urgency and increased urinary frequency, as well as urinary incontinence. In addition, nocturia, which is frequently observed in PwMS, can contribute to decreased sleep quality. Voiding symptoms comprise urinary hesitancy, urinary retention, and the sensation of incomplete emptying. However, there is limited information on the extent to which bladder dysfunction affects perceived sleep disturbances among individuals with MS.

Based on the above, underdiagnosed factors related to sleep disorders among PwMS have become increasingly emphasized nowadays. Our study aims to fill the knowledge gap about the prevalence of sleep disorders in Polish PwMS compared to the healthy population, and also to determine which conditions may affect sleep in this group of patients. In this study, our objective was to evaluate the frequency of sleep disorders in PwMS in comparison to those without the condition. Additionally, we examined the correlation between sleep disorders and symptoms such as fatigue, bladder dysfunction, and mood disorders among PwMS and compared these findings to those without MS.

## 2. Materials and Methods

### 2.1. Study Design and Data Source

This cross-sectional observational study was conducted between July 2020 and August 2023. Participants selected were PwMS between the ages of 18 and 65 who sought treatment at the Neurology Outpatient Clinic within the Department of Neurology at the University Clinical Center, Medical University of Silesia in Katowice, Poland. Finally, a convenience sample of 175 PwMS was enrolled in the study group. We included patients with a diagnosis of MS according to the 2017 McDonald’s criteria and the Expanded Disability Status Scale (EDSS) score < 7.0 (unable to walk 5 m even with aid, essentially uses a wheelchair most of the time; wheels self and transfers alone; up and about in wheelchair some 12 h a day) [10,11]. We aimed to obtain a group which was homogeneous regarding disability. The exclusion criteria were diseases that may affect the quality of sleep (respiratory diseases, heart failure, previously diagnosed mental disorders, abuse of alcohol or hypnotics).

A total of 115 healthy controls, mainly from our clinical centre, were randomly selected and matched for age and gender. The control group consisted of participants who had no history of neurological diseases. The same exclusion criteria as for the study group were applied to participants in the control group.

### 2.2. Ethical Considerations

The Bioethics Committee of the Medical University of Silesia reviewed and approved the study protocol, according to the Declaration of Helsinki (Letter PCN/0022/KB1/46/20). All participants were informed about the study’s aims, and a written informed consent form was taken from each participant.

### 2.3. Study Tools and Outcome Measurements

All participants in this study completed eight questionnaires, including a questionnaire developed by the authors and the Athens Insomnia Scale (AIS), the Epworth Sleepiness Scale (ESS), to assess insomnia and daytime sleepiness, respectively. Additionally, two questionnaires were used to evaluate fatigue: the Fatigue Severity Scale (FSS), the Modified Fatigue Impact Scale (MFIS). To assess pain levels, the Numerical Rating Scale (NRS) was used. The Hospital Anxiety and Depression Scale (HADS) was used to screen for depressive and anxiety disorders. The STOP-Bang questionnaire was used to assess the risk of OSA. The following section provides a description of the questionnaires that were used.

#### 2.3.1. Authors’ Questionnaire

The first questionnaire was used to collect information about the participants. It included demographic data: gender, age, biometric parameters, body mass index = weight/height^2^ (BMI), smoking, education level, habitation, marital status, and professional activity. We also collected medical data: clinical course of MS, DMTs, other chronic diseases, the most disabling symptoms of MS, and types of physical activity. Another part of this form contained questions about subjective sleep disturbances, OSA, RLS, hypnotics used, bladder dysfunction, nocturia, and sleep hygiene. In order to verify the information reported by the patient, data on the onset of the disease, numbers of relapses and the treatment were verified in the patient’s medical history. Healthy controls received a similar questionnaire without questions about MS.

#### 2.3.2. Athens Insomnia Scale

The severity of insomnia was measured by the AIS. It is a brief, self-administered psychometric eight-item tool designed to assess sleep difficulty based on the diagnostic criteria of insomnia of the 10th revision of the international statistical classification of diseases and related health problems (ICD-10) and has been validated in a Polish population with Cronbach’s alpha = 0.89 [12,13]. A cut-off score of 6 or more establishes the diagnosis of border insomnia, and a score over 10 indicates insomnia.

#### 2.3.3. Epworth Sleepiness Scale

The ESS is an eight-item questionnaire that measures subjective sleepiness [14]. It is intended to capture the probability of falling asleep during everyday activities. A score above 10 indicates excessive daytime sleepiness, and a score above 14 indicates pathological sleepiness.

#### 2.3.4. Fatigue Severity Scale and Modified Fatigue Impact Scale

Fatigue was measured using two tools: the FSS and the MFIS. The FSS is the most commonly used nine-item scale to assess fatigue intensity. Participants with a score of more than or equal to 36 are considered to have fatigue [15]. The MFIS consists of 21 items divided into three dimensions of the patient’s quality of life: physical (9 items, Physical MFIS), cognitive (10 items, Cognitive MFIS) and psychosocial (2 items, Psychosocial MFIS). The higher the result, the greater the impact of fatigue on the subject’s functioning (cut-off > 38) [16,17]. The MFIS has been validated for the Polish population [18].

#### 2.3.5. Numerical Rating Scale

The current experience of pain was assessed by the NRS. The respondent rates their pain from 0 to 10, where 0 is “no pain at all”, and 10 is “the worst pain possible” [19]. The NRS is a highly utilized tool due to its significant user friendliness and comprehensibility amongst patients.

#### 2.3.6. Hospital Anxiety and Depression Scale

The HADS is a 14-item self-report instrument for assessing the presence of anxiety (HADS-A) and depressive symptoms (HADS-D) in somatically ill patients [20]. We used Polish translation and validation [21]. The cut-off values for a borderline score were determined for outcomes of 8 or higher, and a score above 11 indicates definitive cases.

#### 2.3.7. The STOP-Bang

The risk of OSA was measured by the STOP-Bang questionnaire [22,23]. It is an 8-item instrument that assesses characteristics known to signal risk for OSA, which form an acronym “STOP-BANG” (Snoring, Tiredness, Observed apneas, high blood Pressure, BMI, Age, Neck circumference, Gender). Scores of three or higher indicate an increased risk of OSA.

#### 2.3.8. RLS

The occurrence of RLS was measured by considering the following statements: (1) the urgency to move the legs, usually accompanied by uncomfortable sensations in the legs; (2) symptoms worsen or are they exclusively present at rest or inactivity; (3) symptoms totally or partially relieved by movement; (4) the symptoms noticed during rest or inactivity get worse or they occur exclusively at night [24]. Subjects had to meet all four core criteria for a strong suspicion of RLS.

#### 2.3.9. Expanded Disability Status Scale

Complete neurological examinations of all patients were carried out, including the EDSS assessment of their disability status.

### 2.4. Sample Size

The sample size calculation was based on the reported prevalence of sleep disorders in PwMS. A similar previous study found that 13.2% of PwMS experienced sleep disturbances, measured and quantified using the AIS, the questionnaire used in this study [25]. A sample size calculation revealed a required sample size of 177 respondents, at a 95% confidence level, and significance level of 0.05.

### 2.5. Statistical Analysis

The statistical analysis was performed using Statistica 13.3 (TIBCO Software Inc. (2017) Statistica (data analysis software system, version 13, http://statistica.io)). Descriptive statistics were used to describe the characteristics of the sample. We present the quantitative variables as mean and standard deviation (SD) or median and interquartile range. The qualitative variables are presented as absolute values and percentages. The normality of distribution was assessed using the Shapiro–Wilk test. As the normal distribution in the analyzed groups was not confirmed, the intergroup differences for the quantitative variable were assessed using the Mann–Whitney U test (variables of skewed distribution). Fisher’s exact test or chi-square test was performed for the qualitative variable. For all the analyses, *p* < 0.05 was considered statistically significant. We applied multivariate logistic regression analysis to assess the impact of different risk factors potentially associated with sleep disorders. Multivariable logistic regression analysis was performed based on the univariate logistic regression results. The best predictive model for sleep disorders in MS was identified. Variables included for multivariate regression were selected from univariate analysis results with *p* < 0.05.

## 3. Results

### 3.1. Comparison between PwMS and Healthy Controls

The study group consisted of 175 participants. The majority of the study group were women (74.86%), and the mean age of the participants was 38.81 ± 11.23 years, with a of range from 18 to 64 years (mean ± SD). The control group comprised 115 healthy controls (HCs), 85 women (73.91%), with a mean age of 38.04 ± 12.10 years. There were significant differences between these groups in education level, earnings, and working status. HCs exhibited a higher level of education than people with multiple sclerosis (PwMS) (76.52% vs. 48.0%, respectively, *p* < 0.001). Moreover, HCs had a better financial situation than PwMS (90.4% vs. 44.%, respectively, *p* < 0.001), and most of them are white-collar workers (56.52% vs. 42.29%, respectively, *p* < 0.001).

Of the 175 PwMS who participated in the study, 157 had relapsing-remitting diagnoses (RRMS), 12 had primary progressive diagnoses (PPMS), and 6 had secondary progressive diagnoses (SPMS). The average disease duration was 7.4 ± 6.21 years. The EDSS score ranged from 0 to 6.5 (mean score 2.41 ± 1.64). In total, 94.29% of PwMS were on a DMT. The most frequent DMTs used were dimethyl fumarate (37.1%), ocrelizumab (10.9%), followed by glatiramer acetate (9.7%), natalizumab (9.7%), and teriflunomide (8%). Detailed characteristics of the study group and the control group are summarized in Table 1.

### 3.2. Sleep Disorders PwMS vs. HCs

In general, individuals with MS experienced subjective sleep disorders more often than the HCs. As shown in Table 2, 67 PwMS (38.3%) and 30 HCs (26.1%) had subjective sleep disturbances (*p* < 0.05). The most frequently reported sleep disorder was insomnia, reported by 25.7% of PwMS and 13% of HCs. Also, PwMS more often used hypnotics than HCs (*p* < 0.001). According to AIS, a trend toward a higher frequency of insomnia was found in 20.6% of PwMS compared with 9.6% HCs (*p* < 0.001).

Subjective excessive daytime sleepiness and pathological sleepiness, as assessed by the ESS, affected 17.1% of PwMS in total. However, the HCs obtained higher results than PwMS in ESS (*p* < 0.001).

In MS group, RLS could be suspected in only 22 out of 175 patients (12.6%). No difference in the occurrence of RLS between the study and the control groups was observed.

A lack of statistical significance was observed when comparing PwMS and the control group in the risk of OSA, according to the STOP-BANG questionnaire. The rate of the PwMS with moderate to high risk of OSA was 10.85% (n = 19).

### 3.3. Bladder Disorders PwMS vs. HCs

Bladder disorders were significantly associated with MS (*p* < 0.001). Almost 40% of PwMS experienced some form of urinary dysfunction. The most common bladder disorders among PwMS were urinary increased frequency (21.6%), urge incontinence (21.6%), urinary retention (16.2%), and urinary hesitation (9.5%). Moreover, nocturia was highly prevalent in our group of PwMS (69.1%). In comparison to the study group, only 10.43% of HCs declared to have some bladder disorders, mainly urinary increased frequency (8 of 115 participants of the control group).

### 3.4. Fatigue, Pain, and Mood Disorders PwMS vs. HCs

In the present study, as reported by the FSS, fatigue was found in 28% of PwMS and in 31.3% of HCs (*p* > 0.05). Moreover, in the second scale measuring fatigue, we did not find any significant differences regarding the total and subscale scores of MFIS. The mean MFIS total score was 29.63 (min: 0, max: 82) in the study group, whereas the mean MFIS total score was 30.3 (min: 0, max: 80) in the control group (*p* > 0.05).

According to the NRS, the mean pain intensity in the study and control groups was 1.88 and 1.59, respectively (*p* > 0.05). This indicates that our groups did not experience severe pain.

Depressive and anxiety disturbances were measured by the HADS. The participants of the control group were more depressed as compared with PwMS (*p* < 0.05). We found that 10.29% and 7.43% of PwMS experienced moderate and severe depressive disturbances, respectively. However, anxiety disorders were more common among PwMS; 13.71% and 14.86% exhibited moderate or severe anxiety, respectively. A lack of statistical significance was obtained when comparing PwMS and the control group by the anxiety subscale of HADS (*p* > 0.05).

Comparisons of the questionnaire results for both groups are given above in Table 2.

### 3.5. Comparison between Female and Male Patients with MS

As shown in Table 3, there were statistically significant differences between women and men with MS. Regarding the assessment of sleep, almost 30% of female MS patients had self-reported insomnia (*p* < 0.05). According to AIS, we found a similar outcome, and insomnia was observed in 25.95% of female PwMS compared to 4.55% of male PwMS (*p* < 0.05). Furthermore, women used more hypnotic medications. Excessive daytime sleepiness was more frequent in women than men (mean score 5.7 vs. 4.2, respectively, *p* < 0.05). The majority of women had a significantly lower risk of OSA in comparison to men, according to the STOP-BANG questionnaire (*p* < 0.05). The increased risk of OSA was mainly observed in male PwMS. The risk of the occurrence of RLS was similar between female and male patients with MS, 12.21% and 13.64%, respectively (*p* > 0.05).

In the MS group, 38.9% women (n = 51) and 40.9% men (n = 18) had some form of bladder disorder (*p* > 0.05). Among women with MS, the most common urinary disorders were urinary frequency (n = 26) and urinary urgency (n = 24).

We also found that fatigue was more frequently observed in women with MS, according to the FSS and MFIS total, physical, psychosocial, and cognitive subscales.

Moreover, women with MS complained of having greater pain.

In addition, female PwMS were more anxious in line with the anxiety subscale of HADS. There were no statistically significant differences between genders in the depression subscale of HADS.

Among PwMS, age and BMI differed significantly between female and male (39.44 ± 11.64 years vs. 36.93 ± 9.8 years and 24 ± 4.8 kg/m^2^ vs. 25.45 ± 3.82 kg/m^2^, respectively; *p* < 0.05).

No significant differences in EDSS score, clinical type of MS, disease onset, disease duration, number of relapses, or MS treatment were found between genders.

Finally, the logistic regression was used to analyze the key factors affecting sleep disorders in PwMS and indicated that female gender (OR = 0.49, 95% CI 0.037–0.94, *p* < 0.05), anxiety disorders (OR = 0.22, 95% CI 0.12–0.32, *p* < 0.001), bladder dysfunction (OR = 0.52, 95% CI 0.16–0.87, *p* < 0.05), were independent predictors of sleep disturbances in PwMS (Table 4).

## 4. Discussion

This study aimed to investigate the prevalence of sleep disturbances, including insomnia, excessive daytime sleepiness, risk of OSA, and risk of RLS, in individuals with MS in comparison to healthy controls. The article also examines common symptoms of MS, such as fatigue, bladder dysfunction, depression, and anxiety, which frequently remain undiagnosed, in addition to the prevalence of sleep abnormalities among PwMS.

In this cross-sectional study, we observed that 20.6% of PwMS experienced insomnia according to the AIS. In comparison, only 9.6% of individuals within the control group reported similar symptoms. Our findings align with the European Sleep Research Society’s report, which presented that approximately 10% of the population suffer from insomnia in industrialized European countries [26]. Zeng et al.’s recent meta-analysis of insomnia prevalence among PwMS revealed a similar prevalence of 22%, as determined by diagnostic tools [5]. Our results are also in accordance with the Portuguese cohort of PwMS in which 22.3% of PwMS fulfilled criteria from The International Classification of Sleep Disorders–Third Edition (ICSD-3) for insomnia [27]. However, some authors revealed a different prevalence ranging between 13.2% and 66.45% of MS patients using the same AIS [25,28,29]. The elevated outcomes may be attributed to self-reported insomnia, which lacks proper verification and standardization [5]. Also, higher frequency in some groups of PwMS is probably because that worse quality of sleep is connected with a higher level of disability caused by EDSS and specific types of treatment [4]. As compared to Pokryszko-Dragan’s study, their cohort of PwMS had a higher rate of insomnia of 49%, a higher EDSS score of 3.2, only 32% of PwMS were treated on interferon (IFN) or glatiramer acetate (GA), and as many as 66% of PwMS did not used DMT [30]. In our study, PwMS showed mild disability with a mean EDSS score of 2.4; what is more, almost all participants received DMT, mainly dimethyl fumarate (DMF), which does not lead to sleep disturbances like IFN or GA [31]. Perhaps due to this, they experienced better sleep quality.

According to the ESS, our sample of PwMS demonstrated both excessive daytime sleepiness (EDS) and pathological sleepiness in 8.57% of cases, resulting in a total prevalence of 17.1%. In other Polish studies, the prevalence of EDS was similar (19%) or significantly higher (41.45%) [29,30]. It is noteworthy that our PwMS exhibited lower levels of EDS compared to the control group, specifically taking into consideration the median ESS score of 5.35 and 7.61, respectively. The control group in our study was randomly selected from a group of medical professionals including physicians, nurses, and physiotherapists, which could have contributed to our observed results. Thus, the results obtained from the ESS may have been impacted by a particular type of work, specifically stressful shift work.

Among PwMS, obstructive sleep apnea (OSA) is the most prevalent form of sleep-disordered breathing. Our observation indicates that nearly 11% of PwMS are at an elevated risk for OSA, with a higher prevalence in males (20.45%). In a recent study of a Croatian cohort, the risk was found to be higher by approximately 29%, primarily in male and older patients [32]. The polysomnographic study revealed that OSA is particularly more prevalent among PwMS who have brainstem lesions, a progressive form of MS, and a lack of DMT [33]. We should note that our results must be interpreted with caution since we utilized the STOP-BANG questionnaire instead of polysomnography, which is the gold standard for diagnosing OSA.

Only 12.6% of our MS patients had a strong likelihood of RLS. This contrasts with a recent meta-analysis in which RLS prevalence was 27.5% (ranging from 13.2% to 65.1%), higher than the HCs [34]. Our findings may be a direct result of modern MS treatment. Currently, we are observing a rapid expansion of novel therapies for MS. The anti-inflammatory effects of DMTs may account for these findings, as inflammation plays a role in one of the theories under genesis of RLS, which is the dysfunction of dopamine systems that leads to RLS. As we mentioned above, most of our study group used DMF as a current MS therapy. Comi et al. obtained results that PwMS on DMF therapy had better sleep quality [31].

This study’s main findings demonstrate that the primary risk factors for developing sleep disorders in individuals with MS were female gender, bladder disorders, and anxiety disorders. It is important to objectively recognize these variables and their potential impact on sleep.

Our study found a greater incidence of sleep disorders among women with MS, consistent with the earlier studies [4,35]. Several factors contribute to a lower quality of sleep in women, including sex hormones, genetic predisposition, fatigue, and mood disturbances (depression and anxiety) [36].

According to our findings, 28% of PwMS report experiencing fatigue as measured by the FSS. We observed a significant discrepancy between males and females, with women experiencing nearly double the level of fatigue compared to men (32.06% vs. 15.91%). Conversely, our findings on fatigue indicate a lower than anticipated outcome, which contrasts with previous studies conducted by us and others [28,37].

The other factors that may play an important role in etiology of sleep disturbances are bladder disorders (storage or voiding problems, or both). They are included in the EDSS examination and can develop at any point during the course of the disease [9,11]. Possible mechanisms for this association include spinal cord lesions leading to bladder dysfunction and the interaction between an overactive immune system and autonomic nervous system [9]. In the current study, 40% of PwMS reported urological symptoms during assessment. Nocturia, which was observed in 69% of PwMS, also contributes to the development of sleep disorders and is linked to sleep deprivation.

Considering the bidirectional effect between bladder dysfunction and mood disorders, there is an increased risk of sleep disorders. Barone et al. demonstrated a bidirectional relationship between mental health and urological symptoms during COVID-19 (the Coronavirus Disease-19) pandemic, when lower urinary tract symptoms (LUTS) were worse in more anxious and stressed individuals [38]. Also, Przydacz et al. found that depressed patients with moderate or severe LUS experienced more sleep disturbances in comparison to individuals with mild LUTS or without any urological problems [39]. Given the complexity of this topic, it is recommended that MS specialists thoroughly examine PwMS for mental health issues and bladder dysfunctions, as these patients are more susceptible to experiencing sleep disorders.

In this study, depressive and anxiety symptoms were more common in people without MS (HCs) than in PwMS. However, gender differences were significant, with women with MS displaying markedly higher levels of anxiety than men. Our research revealed that anxiety contributes significantly to sleep disturbances among PwMS. Similar results were obtained by other researchers [40,41]. The prevalence of anxiety symptoms among PwMS varies in studies, ranging from 15.8% to 57% [42]. People with MS may be more prone to anxiety because of interactions between biological and psychosocial factors. MS-related biological factors, namely changes in brain structure, immune, and inflammatory pathways, may be influential. In addition, psychosocial factors, such as depression, poor coping mechanisms, or inadequate social support, may influence anxiety levels, particularly in women with MS with a younger age of onset, shorter disease duration, and lower disability levels [42].

The impact of sleep disturbance on the progression of MS and on daily functioning in PwMS is currently under investigation. Sahraian et al. explored the potential link between sleep disturbances and an increased risk of acute MS exacerbation due to changes in immune system function. In MS, sleep disorders may lead to an elevated serum level of proinflammatory cytokines, which trigger an autoimmune response. Furthermore, oxidative stress can be toxic for oligodendrocytes and promote the demyelinating process [43]. In another study, PwRRMS who reported poor sleep quality in the previous month were significantly more likely to experience negative disease progression [44]. The study found that relapses of a longer duration were more common, supporting the hypothesis that poor sleep quality may impede recovery from MS relapses, possibly due to a defect in myelin regeneration. These findings suggest that a careful diagnosis of sleep disorders is important in reducing the incidence of acute MS relapses.

Several studies have shown that sleep disturbances have a negative impact on the quality of life of PwMS, particularly in terms of the health-related quality of life (HRQoL). HRQoL measures the extent to which the disease affects the patient’s assessment of their quality of life. Two studies by Veauthier et al. and Kołtuniuk et al. have demonstrated that reduced sleep quality, associated with insomnia and OSA, or insomnia and EDS, respectively, leads to low HRQoL and significantly affects the QoL of PwMS [29,45]. Trojan et al. found in a PSG study that poor sleep quality primarily decreases mental rather than physical HRQoL scores [46]. Similarly, Kotterba et al. indicated that fatigue and poor sleep reduce QoL [40].

These findings should be interpreted in light of several limitations. First, self-reported questionnaires often overestimate the frequency of insomnia, although we utilized the AIS, a validated tool for Polish patients. Second, direct psychiatric evaluation did not confirm depression and anxiety disorders. Additionally, a limited study sample might not be sufficient for accurately representing the broader population of Polish PwMS. The sample was dominated by MS patients with RRMS (89.71%), suggesting this may not be fully representative of the MS population. The exclusion of subjects with an EDSS score of 7 or higher and the relatively low median EDSS score of 2.41 in this MS population limit the generalizability of our results to PwMS with severe disability. Furthermore, participants in the study received diverse forms of DMT. Further research is required with more extensive sample sizes and objective tools to measure the degree of sleep disturbances among Polish individuals with MS.

## 5. Conclusions

Overall, insomnia showed a strong association in patients with multiple sclerosis (PwMS) compared to healthy controls, as revealed by this study. The study also highlighted the connection between sleep disturbances, anxiety, fatigue, and bladder disorders. It is crucial to incorporate assessments of fatigue, bladder dysfunction, and anxiety symptoms as part of the screening process for PwMS at risk of experiencing sleep disturbances. Increasing awareness among neurologists may contribute to early detection and appropriate treatment of sleep disorders, such as pharmacotherapy or cognitive behavioral therapy for insomnia (CBT-I). In addition, we should encourage patients to follow the rules of sleep hygiene to maintain a better quality of sleep. From a neurological perspective, sleep is essential for maintaining the neocortex and consolidating memories. Additionally, quality sleep can enhance immune function due to the strong symbiotic relationship between sleep and the immune system. Chronic sleep impairments have been linked to pro-inflammatory states, which may exacerbate inflammation and contribute to neurodegeneration in MS. Nonetheless, this study can contribute to the ongoing debate about the role of sleep disturbances in PwMS.

## Figures and Tables

**Table 1:** Demographic and clinical characteristics of the studied patients with relapsing-remitting multiple sclerosis (MS), and the control group of healthy volunteers.

Variable	Value				*p*
	MS (n = 175)		HCs (n = 115)		
Gender					0.48
Male, n, %	44	25.14%	30	26.09%	
Female, n, %	131	74.86%	85	73.91%	
Age [years], mean, SD	38.81	11.23	38.03	12.09	0.60
BMI, kg/m^2^, mean, SD	24.38	4.64	25.11	4.34	0.09
Place of residence, n, %					0.39
City < 100 thousands inhibitans	59	33.71%	30	26.09%	
City > 100 thousands inhibitans	77	44.00%	57	49.57%	
Rural areas	39	22.29%	28	24.35%	
Education level, n, %					0.00002
Primary	3	1.71%	1	0.87%	
Secondary	68	38.86%	23	20.00%	
Vocational	20	11.43%	3	2.61%	
Higher	84	48.00%	88	76.52%	
Monthly income [PLN], n, %					<0.001
Under 3000	98	56.00%	11	9.6%	
Over 3000	77	44.00%	104	90.4%	
Working status, n, %					0.00002
White collar	74	42.29%	65	56.52%	
Laborer	25	14.29%	29	25.22%	
Private company owner	9	5.14%	5	4.35%	
Unemployed	10	5.71%	1	0.87%	
Pensioner	34	19.43%	2	1.74%	
Annuitant	8	4.57%	1	0.87%	
Student	12	6.86%	11	9.57%	
No data	3	1.71%	1	0.87%	
Smoking, n, %	31	17.71%	24	20.87%	0.30
Clinical type of MS, n, %					
RRMS	157	89.71%			
SPMS	6	3.43%			
PPMS	12	6.86%			
EDSS, median, min–max	2	0–6.5			
Disease duration, n, %					
<1 year	22	12.57%			
1–5 years	61	34.86%			
6–15 years	69	39.43%			
>15 years	23	13.14%			
Disease Modifying Treatments, n, %					
Dimethyl Fumarate	65	37.14%			
Ocrelizumab	19	10.86%			
Glatiramer acetate	17	9.71%			
Natalizumab	17	9.71%			
Teriflunomide	14	8.00%			
Interferon beta 1a	8	4.57%			
Ofatumumab	6	3.43%			
Cladribine	4	2.29%			
Others DMTs	15	8.58%			
No DMTs	10	5.71%			

Note: BMI, body mass index; DMTs, disease-modifying treatments; EDSS, expanded disability status scale; HCs, healthy controls; MS, sultiple sclerosis; PPMS, primary progressive multiple sclerosis; RRMS, relapsing-remitting multiple sclerosis; SPMS, secondary progressive multiple sclerosis.

**Table 2:** Sleep and scale characteristics of the studied patients with relapsing-remitting multiple sclerosis (MS), and the control group of healthy volunteers.

Variable	Value				*p*
	MS (n = 175)		HCs (n = 115)		
Subjective sleep disorders, n, %					
Normal sleep	103	58.86%	80	69.57%	
Insomnia	45	25.71%	15	13.04%	0.014
Hypersomnia	22	12.57%	15	13.04%	
Hypnotics, n, %	27	15.43%	4	3.48%	0.0007
Prescription medicines	13	7.43%	2	1.74%	0.0055
Over-the-counter medicines	14	8.00%	2	1.74%	
Bladder disorders, n, %	69	39.43%	12	10.43%	<0.001
AIS					
Normal sleep, n, %	98	56.00%	50	43.48%	<0.001
Borderline insomnia, n, %	41	23.43%	54	46.96%	
Insomnia, n, %	36	20.57%	11	9.57%	
ESS					
ESS score, mean, SD	5.35	4.69	7.61	3.92	0.000002
Normal sleep, n, %	145	82.86%	90	78.26%	0.47
Excessive daytime sleepiness, n, %	15	8.57%	15	13.04%	
Pathological sleepiness, n, %	15	8.57%	10	8.70%	
Risk of RLS, n, %	22	12.57%	16	13.91%	0.44
Risk of OSA, STOP-BANG, n, %					
Low risk	156	89.14%	95	82.61%	0.09
Moderate risk	17	9.71%	20	17.39%	
High risk	2	1.14%	0	0.00%	
FSS, n, %					
Fatigue	49	28.00%	36	31.30%	0.32
Non-fatigue	126	72.00%	79	68.70%	
MFIS, mean, SD					
MFIS total score	29.63	20.73	30.31	15.56	0.47
MFIS Cognitive subscale	13.25	9.86	14.6	7.82	
MFIS Psychosocial subscale	2.68	2.8	3.07	1.98	
MFIS Physical subscale	13.7	9.98	12.49	7.38	
NRS, mean, SD	1.88	2.48	1.59	2.02	0.65
HADS-Anxiety, mean, SD	6.18	3.84	6.88	3.97	0.08
Normal, n, %	125	71.45%	72	62.61%	
Borderline anxiety, n, %	24	13.71%	26	22.61%	
Anxiety, n, %	26	14.86%	17	14.78%	
HADS-Depression, mean, SD	4.01	3.78	4.75	3.46	0.023
Normal, n, %	144	82.29%	92	80%	
Borderline depression, n, %	18	10.29%	16	13.91%	
Depression, n, %	13	7.43%	7	6.09%	

Note: AIS, Athens Insomnia Scale; ESS, Epworth Sleepiness Scale; FSS, Fatigue Severity Scale; HADS, Hospital Anxiety and Depression Scale; HCs, healthy controls; MFIS, Modified Fatigue Impact Scale; MS, multiple sclerosis; NRS, Numerical Rating Scale; OSA, obstructive sleep apnea; RLS, restless legs syndrome.

**Table 3:** Sleep disorders, fatigue, bladder disorders, and mood disorders among female and male patients with multiple sclerosis.

Sleep Disorders among Patients with Multiple Sclerosis					
	Gender				
	Female (n = 131)		Male (n = 44)		*p*
Self-reported sleep disorders, n, %					
Normal sleep	68	51.91%	35	79.55%	0.005
Insomnia	39	29.77%	6	13.64%	
Hypersomnia	20	15.27%	2	4.55%	
Hypnotics, %	25	19.08%	2	4.55%	0.013
Prescription medicines	12	9.16%	1	2.27%	0.069
Over-the-counter medicines	13	9.92%	1	2.27%	
History of using sleeping medications, n, %	10	7.63%	4	9.09%	0.49
Bladder disorders, n, %	51	38.93%	18	40.91%	0.48
AIS, n, %					
Normal sleep	67	51.15%	31	70.45%	0.008
Borderline insomnia	30	22.90%	11	25.00%	
Insomnia	34	25.95%	2	4.55%	
Risk of RLS, n, %	16	12.21%	6	13.64%	0.49
Risk of OSA, STOP-BANG, n, %					
Low risk	121	92.37%	35	79.55%	0.016
Moderate risk	8	6.11%	9	20.45%	
High risk	2	1.53%	0	0.00%	
ESS, n, %					
ESS total score, mean, SD	5.74	4.86	4.2	3.94	0.011
Normal sleep	105	80.15%	40	90.91%	0.18
Excessive daytime sleepiness	12	9.16%	3	6.82%	
Pathological sleepiness	14	10.69%	1	2.27%	
FSS, n, %					
Fatigue	42	32.06%	7	15.91%	0.027
Non-fatigue	89	67.94%	37	84.09%	
MFIS, mean, SD					
MFIS Total score	32.5	20.94	21.1	17.71	0.0009
MFIS Cognitive subscale	14.39	10.22	9.86	7.87	0.011
MFIS Psychosocial subscale	3.04	2.29	1.64	1.89	0.0011
MFIS Physical subscale	15.08	9.92	9.57	9.05	0.00024
NRS, mean, SD	2.12	2.63	1.16	1.8	0.0027
HADS, mean, SD					
HADS-Anxiety subscale	6.8	3.83	4.3	3.25	0.00019
HADS-Depression subscale	4.25	3.82	3.25	3.57	0.16

Note: AIS, Athens Insomnia Scale; ESS, Epworth Sleepiness Scale; FSS, Fatigue Severity Scale; HADS, Hospital Anxiety and Depression Scale; HADS-A, Hospital Anxiety and Depression Scale-Anxiety Subscale; HADS-D, Hospital Anxiety and Depression Scale-Depression Subscale; MFIS, Modified Fatigue Impact Scale; NRS, Numerical Rating Scale; OSA, obstructive sleep apnea; RLS, restless legs syndrome.

**Table 4:** The logistic regression model of predictors of sleep disorders among patients with multiple sclerosis.

Covariate	OR	95% CI		*p*
		95%−	95%+	
Anxiety disorder (HADS-A)	0.22	0.12	0.32	0.00003
Bladder disorders	0.52	0.16	0.87	0.004
Female Gender	0.49	0.037	0.94	0.03

Note: CI, confidence interval; HADS-A, Hospital Anxiety and Depression Scale-Anxiety Subscale; OR, Odds Ratio.

## Data Availability

The data presented in this study are available from the corresponding author upon request. The data are not publicly available due to law restrictions [any medical data are sensitive information].
